# Effect of Maternal Triclosan Exposure on Neonatal Birth Weight and Children Triclosan Exposure on Children's BMI: A Meta-Analysis

**DOI:** 10.3389/fpubh.2021.648196

**Published:** 2021-07-08

**Authors:** Jiani Liu, Danrong Chen, Yanqiu Huang, Francis Manyori Bigambo, Ting Chen, Xu Wang

**Affiliations:** ^1^State Key Laboratory of Reproductive Medicine, Center for Global Health, School of Public Health, Nanjing Medical University, Nanjing, China; ^2^Key Laboratory of Modern Toxicology of Ministry of Education, School of Public Health, Nanjing Medical University, Nanjing, China; ^3^Women's Hospital of Nanjing Medical University, Nanjing Maternity and Child Health Care Hospital, Nanjing, China; ^4^Department of Endocrinology, Children's Hospital of Nanjing Medical University, Nanjing, China

**Keywords:** environmental, urinary, pregnancy, neonatal, epidemiological

## Abstract

**Background:** Triclosan (TCS) is an environmental chemical with endocrine disrupting effects and can enter the body through the skin or oral mucosa. Human data about the effect of TCS exposure during pregnancy on neonatal birth weight and TCS exposure during childhood on children's growth are scarce.

**Objectives:** To investigate the association between maternal urinary TCS level and neonatal birth weight, as well as children's urinary TCS level and children's body mass index (BMI).

**Methods:** A systematic literature search was conducted using PubMed, Cochrane Library, and Web of Science. Finally, seven epidemiological articles with 5,006 participants from September 25, 2014 to August 10, 2018 were included in the meta-analysis to identify the relationship between maternal exposure to TCS and neonatal birth weight. On the other hand, three epidemiological articles with 5,213 participants from July 22, 2014 to September 1, 2017 were included in another meta-analysis to identify the relationship between children's exposure to TCS and children's BMI. We used Stata 16.0 to test the heterogeneity among the studies and calculating the combined effect value 95% confidence interval (CI) of the selected corresponding models.

**Results:** TCS exposure during pregnancy was not significant associated with neonatal birth weight. The results of forest plots were as follows: ES (Estimate) = 0.41 (95% CI: −11.97–12.78). Children's urinary TCS level was also irrelevant associated with children's BMI: ES = 0.03 (95% CI: −0.54–0.60).

**Conclusions:** This meta-analysis demonstrated that there was no significant association between maternal TCS level and neonatal birth weight, also there has no relationship between children's urinary TCS level and children's BMI.

## Introduction

Triclosan (TCS) is a synthetic, broad-spectrum biocides, and it was first licensed for use in the 1960s (1). Nowadays, TCS is still widely used as an important antibacterial in consumer products such as soap, hand sanitizer, toothpaste, and mouthwash. TCS can be absorbed into the human body, then enter various human fluids and tissues from skin or oral mucosa (2).

Currently, the relationship between exposure to TCS during pregnancy and neonatal birth weight as well as children's body mass index (BMI) is not clear. A previous study has found that females tended to have higher TCS exposure level than males (3). A study of mothers in New York found that TCS could be detected in 100% of the 181 urine samples (4). In pregnant women, TCS in maternal serum can reach the fetus through the placental barrier, and it can be detected in umbilical cord blood (5). According to some researches, maternal exposure to TCS during pregnancy can cause certain effects on neonatal birth outcomes, such as head circumference, birth weight, and birth length (6–8). Among them, abnormal birthweight is an important birth outcome and will lead to a series of consequences. Babies born with low birthweight will face increased risks for adverse health effects in a lifetime, including cerebral palsy, neurological disabilities, and vision or hearing impairment (9). Whereas, those who are born with higher birthweight are more likely to develop obesity and breast cancer (10–13). Some studies have suggested that maternal TCS exposure can lead to increased (14) or decreased (6, 7, 15, 16) birth weight. However, some articles have shown that there was no significant relationship between maternal TCS exposure and neonatal birth weight (17–20). In addition, children are also exposed to TCS during childhood (21), and it was found that TCS exposure during this period may be related to children's growth development (16, 22). Childhood obesity is an important risk factor for children's health. In addition to the psychological consequences, obesity also increases the risk of type 2 diabetes mellitus, hyperlipidemia, hypertension, cardiovascular disease, sleep apnea, cancer, and arthritis (23). But the relationship between children's TCS exposure level and children's BMI remains controversial (22, 24).

Therefore, exploring the effects of exposure to TCS during pregnancy on neonatal birthweight and exposure to TCS during childhood on children's BMI are priorities. However, there is lack of a systematic studies about the topic. The purpose of our research is mainly to compare the effects of TCS exposure on growth indicators before and after birth. We also explored the heterogeneity of the included studies and make a subgroup analysis of involved factors that may affect the outcomes.

## Materials and Methods

### Search Strategy

In our study, we used the following search terms to retrieve the relevant literature in three electronic bibliographic databases: PubMed, Cochrane Library, and Web of Science.

#### TCS Exposure During Pregnancy on Neonatal Birth Weight

In this direction, we searched the published literature until December 2019.

#1: (gestation) or (pregnancy) or (prenatal) or (antepartum) or (maternal)
#2: (triclosan) or (TCS) or (phenols)
#3: (birth weight) or (neonatal weight) or (baby weight) or (birthweight) or (birth size) or (fetal growth)
#4: #1 AND #2 AND #3

#### TCS Exposure During Childhood on Children's BMI

In this direction, we searched the published literature until December 2019.

#1: (triclosan) or (TCS)
#2: (obesity) or (fat) or (plump) or (obese) or (corpulent) or (overweight) or (weight) or (axunge)
#3: #1 AND #2

### Inclusion Criteria

#### TCS Exposure During Pregnancy on Neonatal Birth Weight

The inclusion criteria for the studies were:

- Able to search the full text of literature;
- Pregnant women as the subject, not experimental animals;
- Explicitly specified the substance that pregnant women were exposed to TCS during pregnancy;
- The data showing the correlation between TCS concentration and neonatal birth weight were provided, such as estimate (ES) and 95% confidence interval (95% Cl);
- If the same population was used in different studies, we selected the recent one with larger sample size;
- The research design had no defects and the literature quality was high.

#### TCS Exposure During Childhood on Children's BMI

The inclusion criteria for the studies were:

- Able to search the full text of literature;
- Infants or children as the subjects, not experimental animals (population);
- Explicitly specified the substance that infants or children were exposed to TCS (ES);
- The data showing the correlation between TCS concentration and children BMI were provided, such as ES and 95% CI (outcome);
- If the same population was used in different studies, we selected the recent one with larger sample size;
- The research design had no defects and the literature quality was high.

### Exclusion Criteria

The exclusion criteria for the studies were:

(1) Not conform to the research topic;
(2) Animal studies, conference abstract, lecture literature, editorial materials or comments, and so on;
(3) Studies had design defects and poor quality;
(4) The research objects exposure to multiple environmental endocrine disruptors;
(5) Raw data was unavailable;
(6) Unpublished studies.

### Study Selection and Data Extraction

At first, the titles and abstracts of all identified publications were independently reviewed by two authors based on the inclusion criteria and exclusion criteria for the eligibility of the included articles. Then, further screening of the remaining full retrieved papers were implemented, and the references of the selected articles were evaluated. We identified the literature that were finally adopted. Studies were exported in Endnote X7, and duplicates were automatically removed. To ensure the accuracy of the results, the two authors have also checked and corrected for duplicates manually. According to literature inclusion criteria, we used a predefined template to extract information (25). Any disagreement of between the two assessors in any of the above was settled by a discussion with a third evaluator. Then, we extracted the information of each literature, including author name, publication time, study design, location, sample size, outcome, exposure distribution, effect size, and covariate adjustment in our predesigned spreadsheet.

### Quality Evaluation

Referring to the Newcastle Ottawa Scale (NOS) standard, the two authors independently evaluated the quality of each literature (26). NOS consists of three categories (selection of subject, comparability, and outcomes) and eight items. NOS ranges from 0 to 9 stars: 4 stars for selection, 2 stars for comparability, and 3 stars for outcomes. We rated each study based on criteria to estimate whether it can be included in the study. If the total number of stars is ≥6, we consider the research quality to be high. If the total number of stars is 3–5, we consider the research quality to be moderate. Otherwise, the research quality is too low and was excluded (27). The disputes regarding the grading of the literature were discussed and resolved together.

### Statistical Method

All data acquisition and analysis were processed in the software Stata 16.0. We inserted appropriate information extracted from related literature in the spreadsheets and used the meta-analysis module to perform statistical analysis. We used the adjusted 95% CI in both birth weight and children's BMI as the effect value for TCS exposure during pregnancy on neonatal birth weight and children TCS exposure on children's BMI, respectively.

Specific steps: (1) Subgroup analysis: We explore the characteristics of the original studies and performed a hierarchical analysis based on different adjusted methods of TCS, different gender of infants, and different trimester that TCS was detected, and calculate the 95% CI of each subgroup; (2) Sensitivity analysis: We used a one-by-one elimination method for sensitivity analysis and chose a random effects model to analyze potential instability factors in meta-analysis, and evaluate the impact of publication bias of the overall results; (3) Heterogeneity test: If *P* < 0.05, *i*^2^ ≥ 50%, the articles were regarded to have high heterogeneity. Considering that heterogeneity will drive different statistical methods to summarize the data, if heterogeneity is expected, random effects models will be preferable to fixed effects models (28). Otherwise, we will choose fixed effects models; (4) Test of publication bias: Begg's funnel chart and Egger's method are used to qualitatively and quantitatively test and evaluate the publication bias of literature data. The funnel plot should be similar to the asymmetrical inverted funnel, and the dispersion of small samples is large, so it is often at the bottom of the funnel chart, and the dispersion of large samples is small, so it is at the top. Egger's method is used to evaluate whether there is asymmetry related to standard error in the research results. Since the number of our studies included <10, we use only Begg's funnel plots to analyze publication bias.

## Results

### Study Search and Characteristics Overview

#### TCS Exposure During Pregnancy on Neonatal Birth Weight

**Figure 1:**
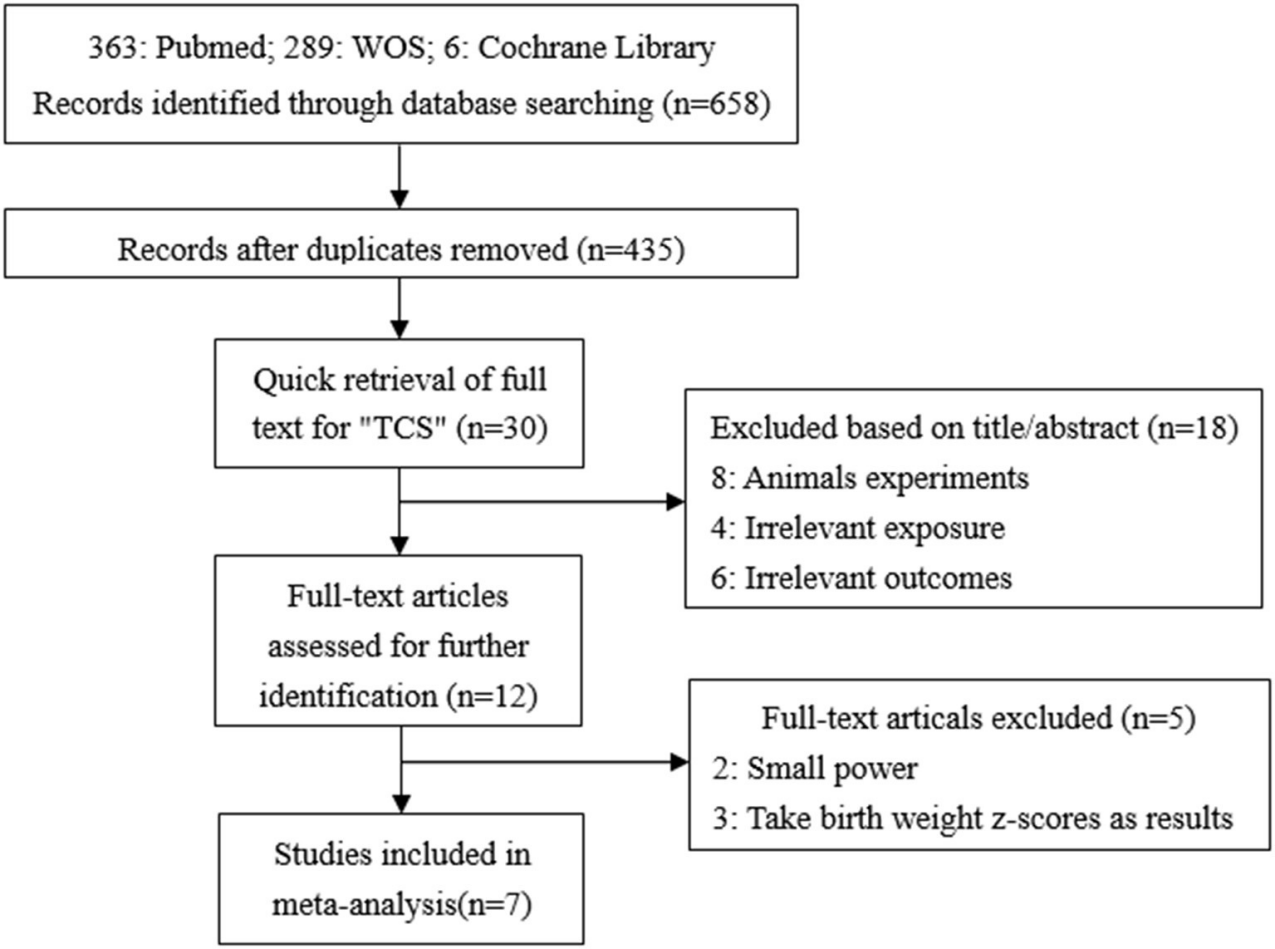
Flow chart of the association between maternal urinary TCS level and neonatal birth weight.

A total of 658 studies were initially included, and 435 records were obtained after eliminating the duplicate. By searching for the keyword (TCS) of the full text, 30 records were remaining. By reading titles and abstracts, 10 records were excluded due to irrelevant exposures or outcomes and eight records were experimenting on animals. The remaining 12 records were selected through reviewing the full papers. Among 12 full papers selected, five records were not considered since two had too small a sample size, and the other three records took the change in z-values as the results. Finally, seven articles were identified and included in our study (7, 14, 15, 17–20) (Figure 1). All seven studies used concentrations of urinary TCS as exposure factor, and also four of these studies used creatinine-corrected as the adjusted methods of TCS; the others used specific gravity corrected. Additionally, two out of seven studies analyzed male and female infants separately, so we have nine sets of data.

#### TCS Exposure During Childhood on Children's BMI

**Figure 2:**
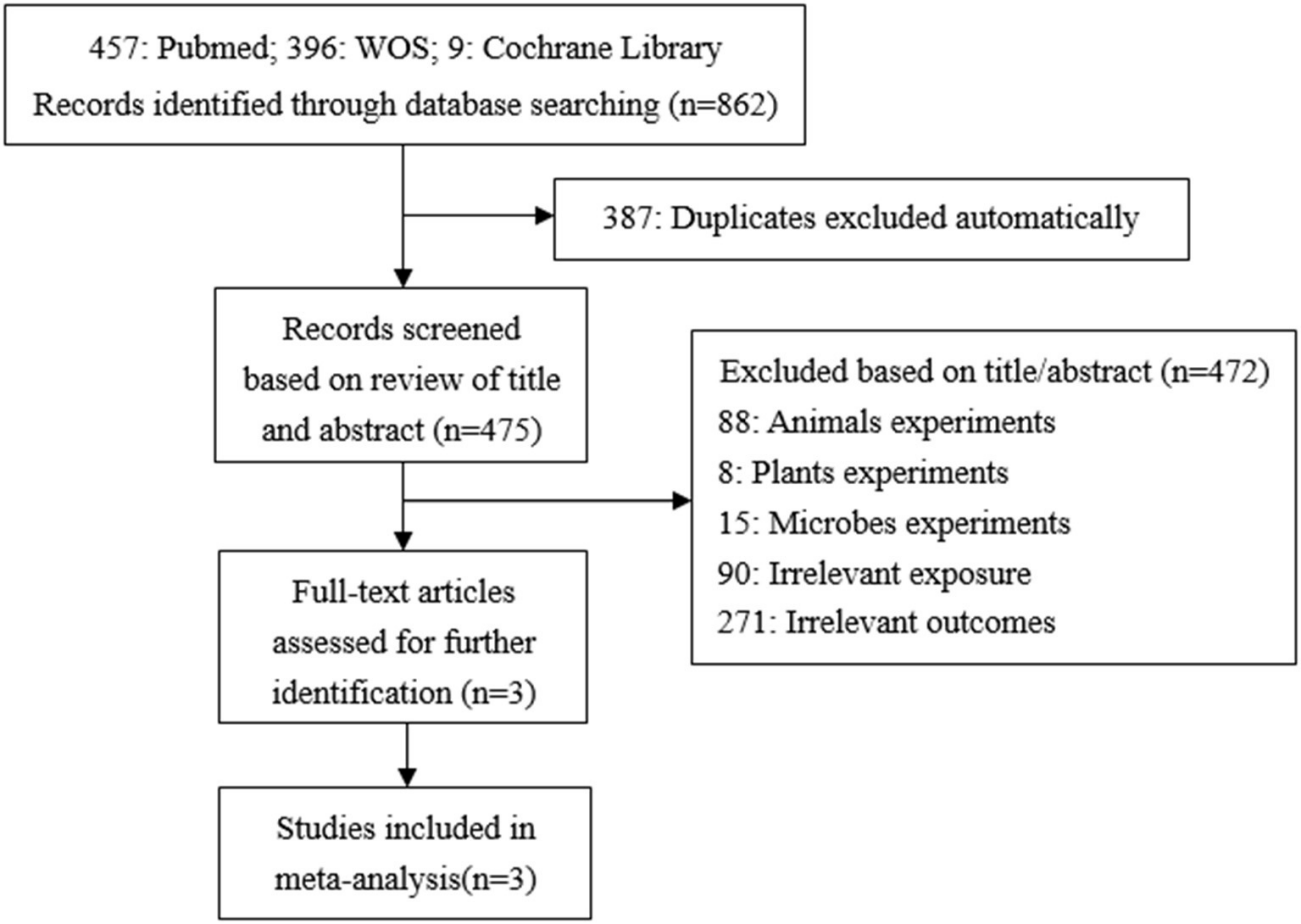
Flow chart of the association between urinary TCS level in children and children's BMI.

A total of 862 studies were initially included, and 475 records were obtained after eliminating the duplicate. By reading titles and abstracts, 361 records were excluded due to irrelevant exposures or outcomes, 88 records were experimenting on animals, eight records on plants, and 15 records on microbes. Finally, three articles were identified and included in our study (29–31) (Figure 2). In the same way, all of these studies used concentrations of urinary TCS as exposure factor, and the adjusted methods of TCS were creatinine-corrected.

### Quality Evaluation

The methodological quality of included studies was assessed by using the NOS. The characteristics of these studies are displayed in Table 1, and all of them are cohort studies. The quality assessment score of our included studies range from 6 to 8, and we considered the research quality to be high.

**Table 1:** Main characteristics of studies included in the meta-analysis.

**First author name**	**Country**	**Publication year**	**Research design**	**Sample size**	**Adjusted methods of TCS**	**Outcome (95% CI)**	**Adjusted variable**	**Quality assessment score**
**TCS exposure during pregnancy on neonatal birth weight**								
Philippat, C	France	2014	Cohort	520	Urine creatinine	4.60 (−49.00, 58.30)	Maternal and paternal height, pre-pregnancy weight, maternal active and passive smoking during pregnancy, maternal education level, gestational age at measurement and parity	8
Geer, L. A	USA	2017	Cohort	185	Urine creatinine	−89.37 (−373.83, 195.08)	Maternal age, nativity, and neonate gender	8
Ding, G	China	2017	Cohort	496	Urine creatinine	72.79 (−35.38, 180.96)	Maternal age, pre-pregnancy BMI, weight gain during pregnancy, passive smoking, household monthly income, infant gender, gestational age, and parity	7
Ouyang, F	China	2018	Cohort	620	Urine creatinine	−88.70 (−190.60, 13.30) 41.90 (−64.80, 148.50)	Urinary creatinine, maternal age, education, passive smoking, parity, and pre-pregnancy BMI categories	8
Messerlian, C	USA	2018	Cohort	346	Specific gravity (SG)	−16.00 (−54.00, 23.00)	Maternal age, maternal BMI, maternal education, maternal smoking, IVF (*in-vitro* fertilization)-based vs. non-IVF based treatment and season	7
Huo, W	China	2018	Cohort	1,006	Specific gravity (SG)	1.54 (−16.23, 19.30) −10.99 (−28.18, 6.20)	Infant's sex, maternal age, pre-pregnancy BMI, maternal education, parity, delivery mode, gestational age, and passive smoking during pregnancy	8
Lester, F	Canada	2018	Cohort	1,833	Specific gravity (SG)	11.85 (0.06, 24.70)	Mother's age, BMI, education level, nulliparity status, place of birth, income level, and smoking status	7
**TCS exposure during childhood on children's BMI**								
Buser, M. C	USA	2014	Cohort	1,298	Urine creatinine	0.12 (−0.12, 0.36)	Age, sex, race/ethnicity, calorie intake, television and video game use (6–11 y), recreational activity (12–19 y), serum cotinine, income level, and urinary creatinine	8
Li, S	USA	2015	Cohort	2,898	Urine creatinine	−0.47 (−0.69, −0.26)	Sex, age, race/ethnicity, poverty to income ratio, cotinine levels, and urinary BPA concentrations	8
Deierlein, A. L	USA	2017	Cohort	1,017	Urine creatinine	0.89 (−0.08, 1.86)	Race, age, educational level, socioeconomic status, baseline BMI	7

### Correlated Forest Plots

#### Relationship Between Maternal Urinary TCS Level and Neonatal Birthweight

**Figure 3:**
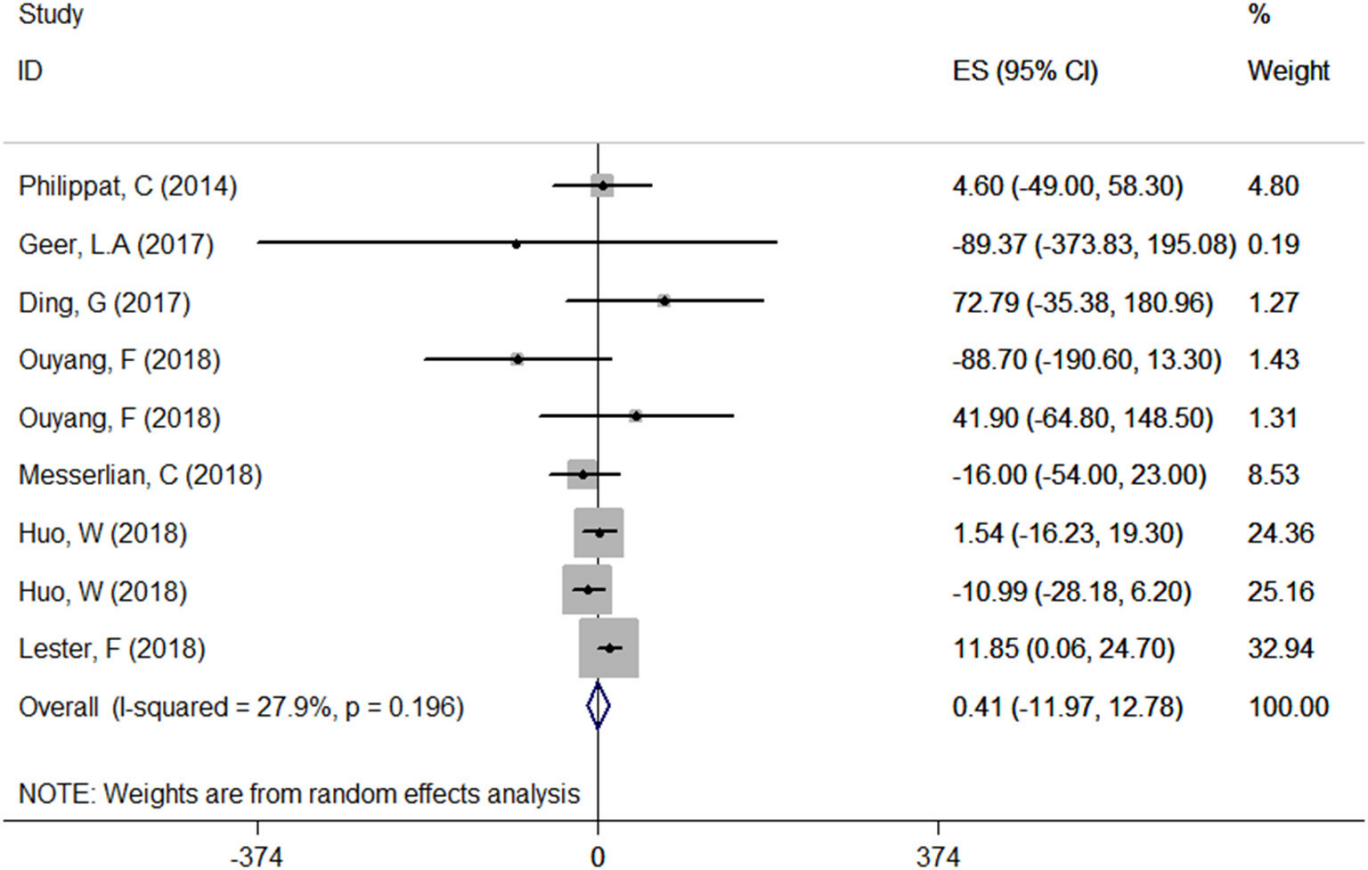
Forest plot of TCS exposure during pregnancy and neonatal birth weight.

A total of seven articles were combined to analyze the relationship between TCS exposure during pregnancy and neonatal birth weight. Among seven articles, two articles studied male and female infants separately, so we have nine sets of data. The forest plot results showed that only one article (20) indicated that exposure to TCS during pregnancy may lead to the increase of neonatal birth weight with ES = 11.85 (95% CI: 0.06–24.70), while other studies found no relationship. Specfic results were as follows: ES = 0.41 (95% CI: −11.97–12.78), *P* = 0.196, *i*^2^ < 50%. The forest plot graph (Figure 3) shows that there are large overlaps in the CIs of these studies, indicating little heterogeneity among these studies. And there was no significant association between maternal urinary TCS level and neonatal birth weight.

#### Relationship Between Children's Urine TCS Level and BMI

**Figure 4:**
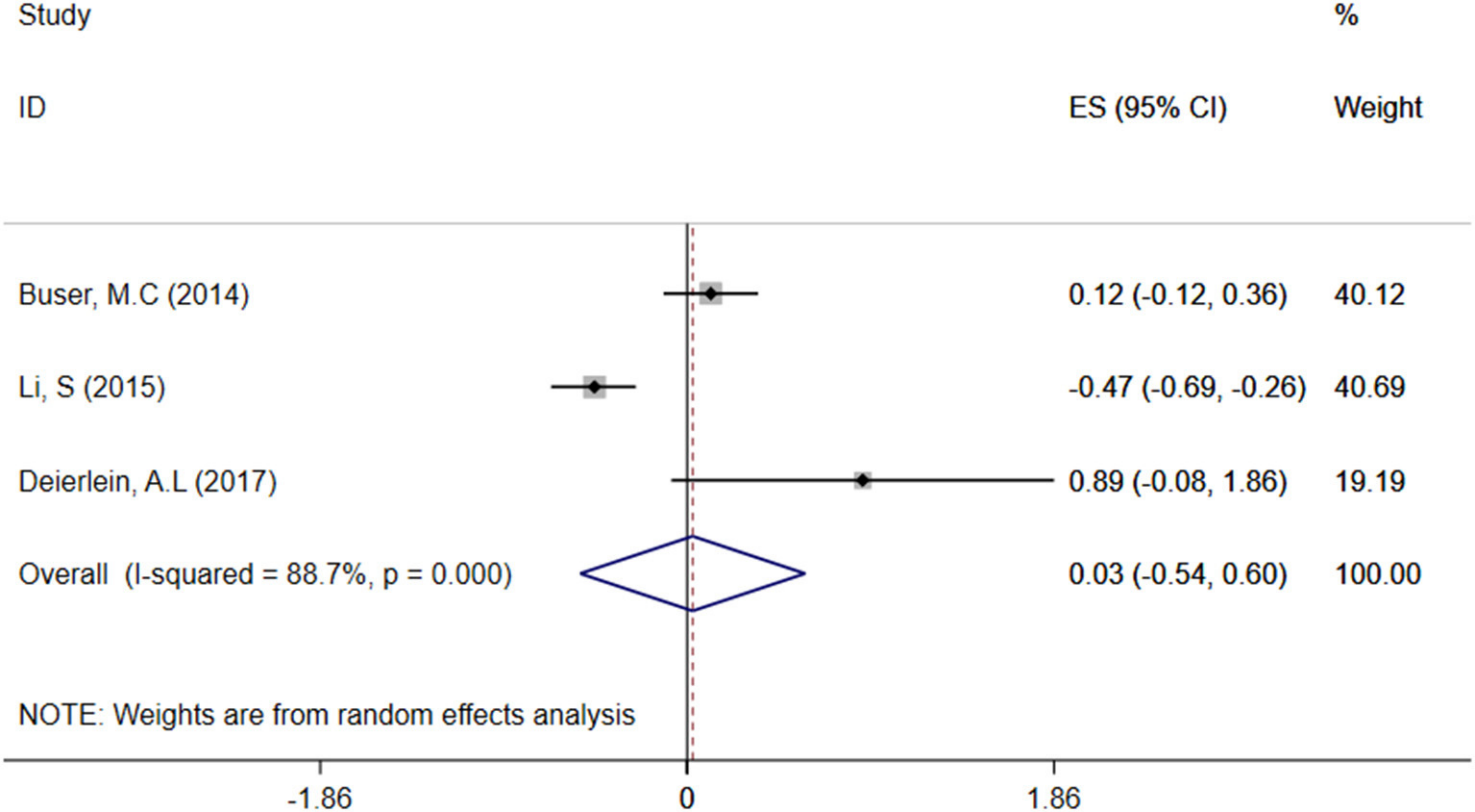
Forest plot of TCS exposure during childhood and children's BMI.

A total of three articles were combined to analyze the relationship between TCS exposure during childhood and children's BMI. TCS was adjusted for urine creatinine in all studies. Three articles identified a irrelevant relationship between TCS exposure during childhood and children's BMI (29–31). Specific results were as follows: ES = 0.03 (95% CI: −0.54–0.60), *P* = 0.000, *i*^2^ ≥ 50%, indicating a great heterogeneity among these studies. And there had no association between TCS exposure during childhood and children's BMI (Figure 4).

### Subgroup Analysis—The Relationship Between Exposure to TCS of Different Adjusted Methods During Pregnancy and Neonatal Birth Weight

**Figure 5:**
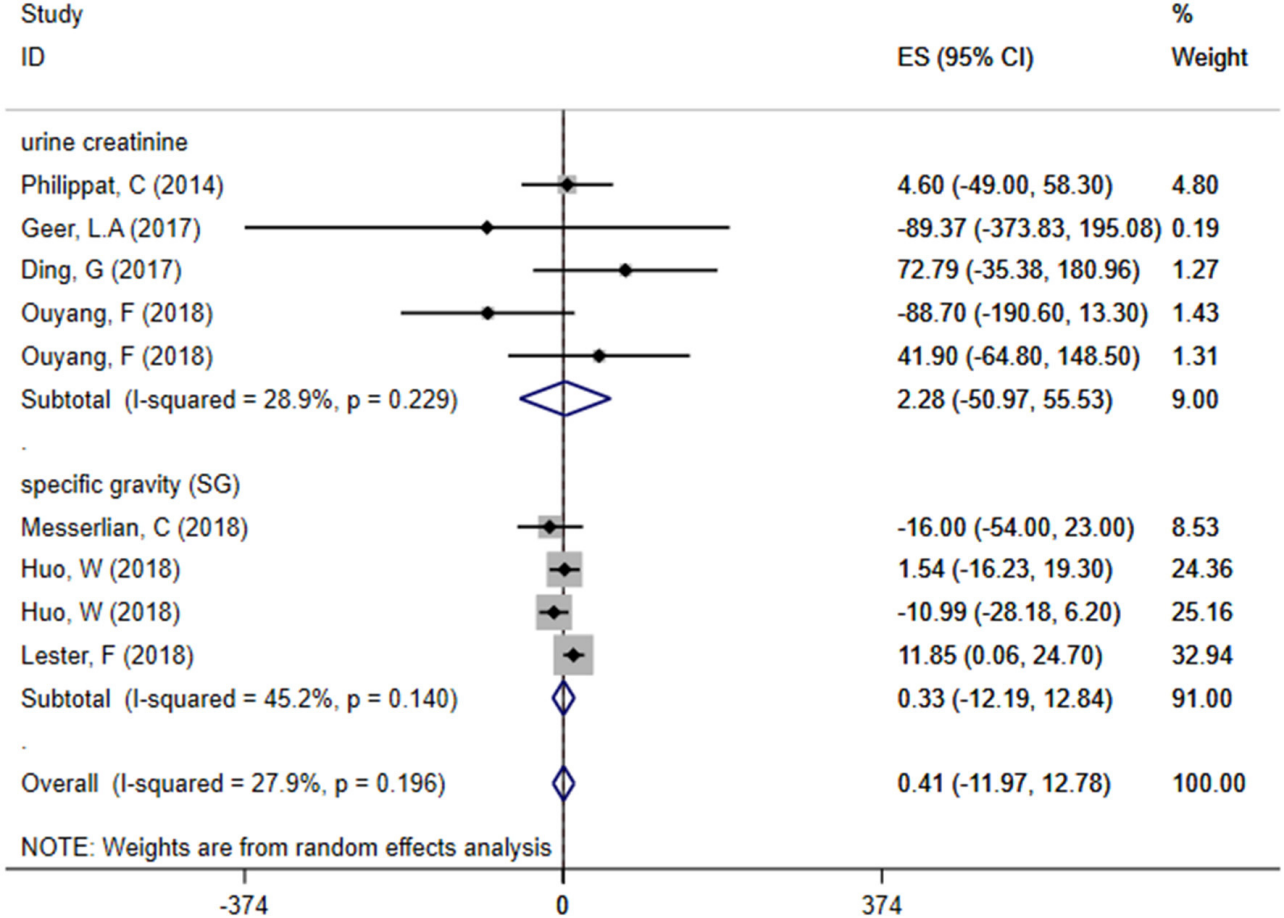
Subgroup analysis of different adjusted methods of TCS in TCS exposure on neonatal birth weight.

We divided included studies into two groups based on the adjusted methods of TCS of either urine creatinine or specific gravity (SG). The results were as follows: ES = 2.28 (95% CI: −50.97–55.53), *P* = 0.229, *i*^2^ < 50% and ES = 0.33 (95% CI: −12.19–12.84), *P* = 0.140, *i*^2^ < 50%, respectively, which manifested that whether use urine creatinine or SG as the adjusted method of TCS, the maternal urinary TCS level had no significant effect on neonatal birth weight. Meta-analysis forest plot was shown in Figure 5.

### Sensitivity Analyses

**Figure 6:**
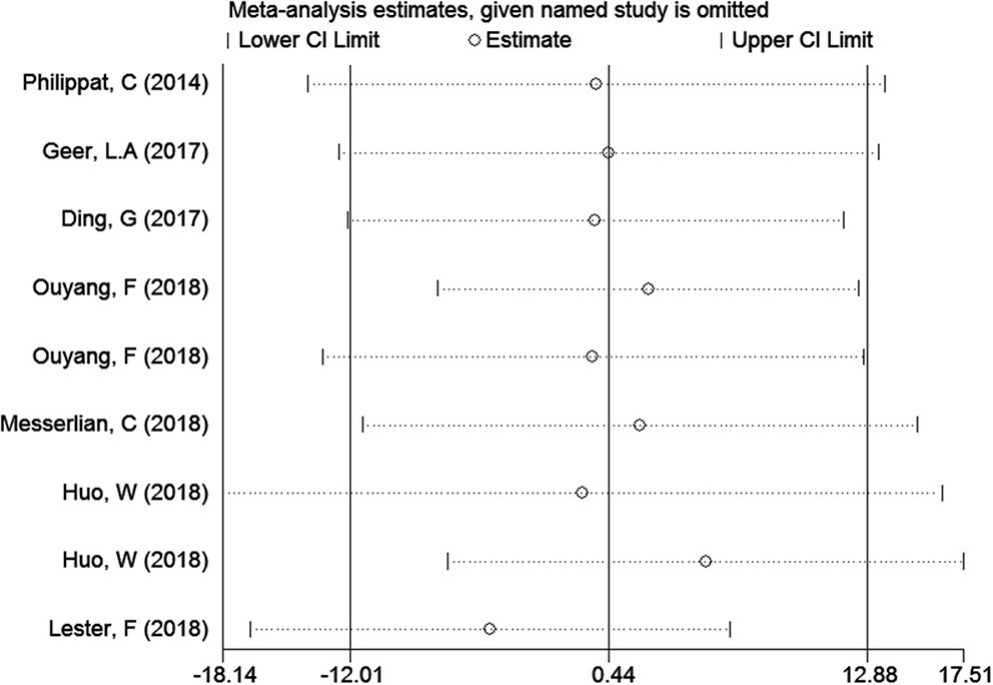
Sensitivity analysis of TCS exposure during pregnancy and neonatal birth weight.

**Figure 7:**
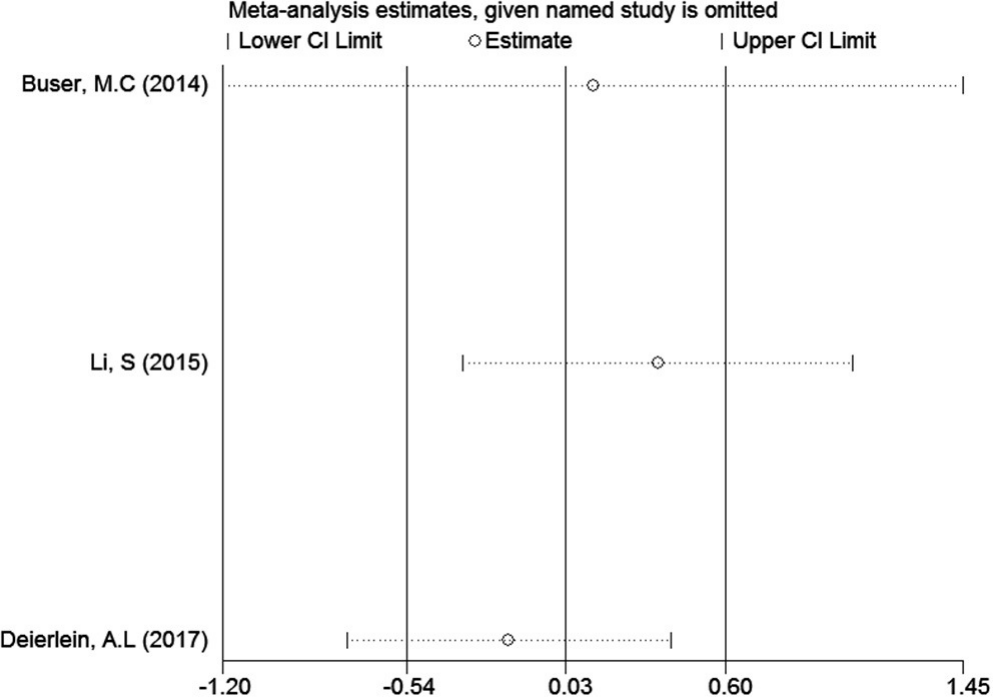
Sensitivity analysis of TCS exposure during childhood and children's BMI.

In the meta-analysis of the association between maternal urinary TCS level and neonatal birth weight as well as urinary TCS level of children and children's BMI, several articles were combined, which may lead to heterogeneity between studies. However, the results showed that the pooled ES values before and after the exclusion of a study were essentially the same as the 95% CIs, indicating that the original meta-analysis was reliable (Figures 6, 7).

### Publication Bias

**Figure 8:**
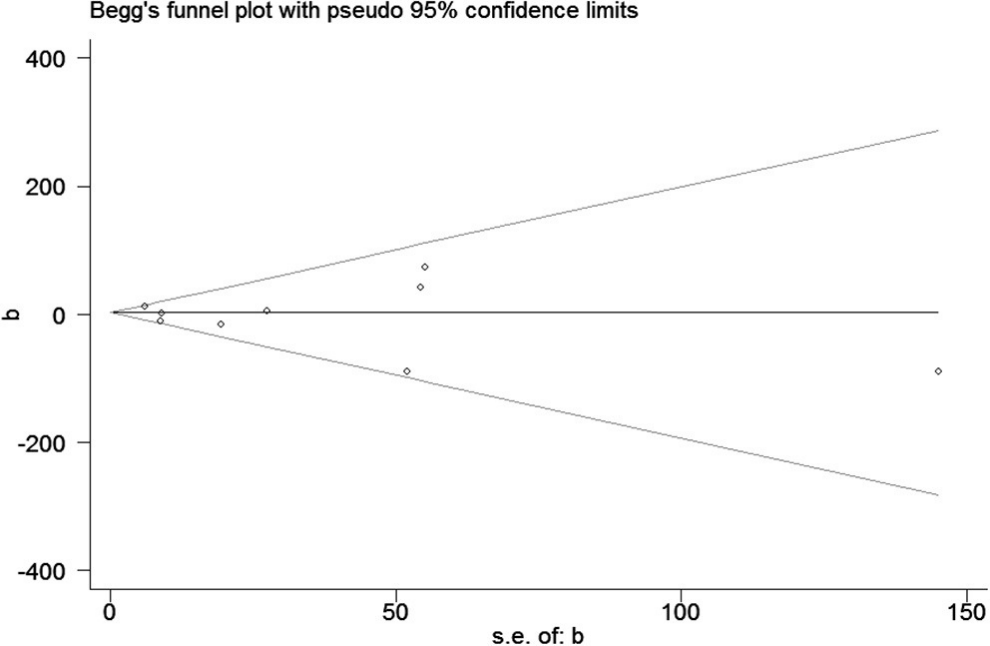
The funnel plot of TCS exposure during pregnancy and neonatal birthweight.

**Figure 9:**
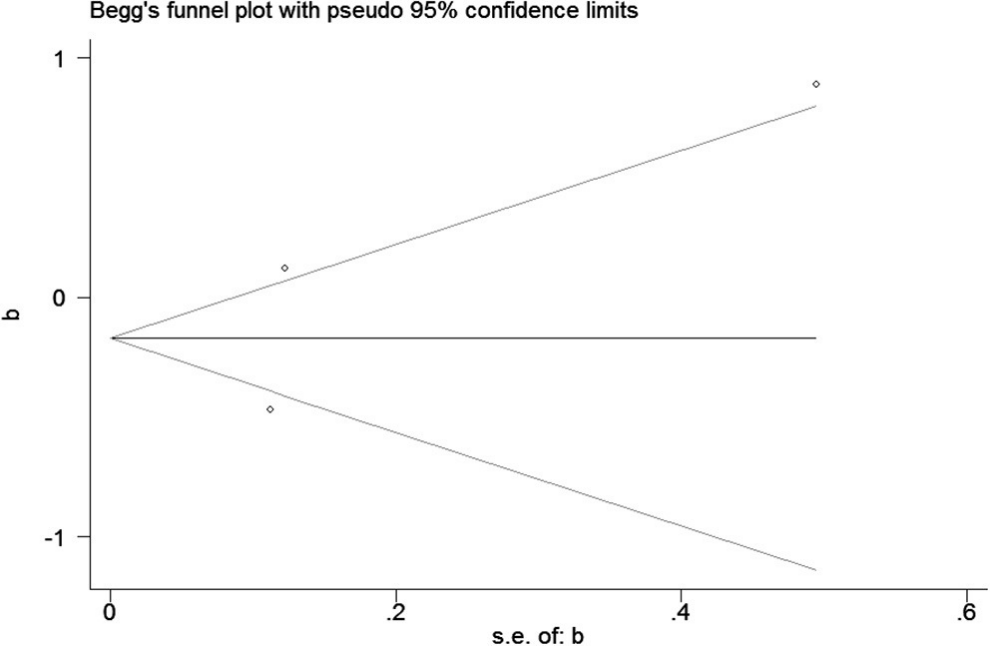
The funnel plot of TCS exposure during childhood and children's BMI.

Publication bias was observed for TCS exposure during pregnancy and birth weight as well as TCS exposure during childhood and children's BMI using Begg's test and funnel plots (*P* = 0.754; Figure 8 and *p* = 1.000; Figure 9). In Figure 9, all three points fall outside, suggesting the possibility of heterogeneity, but too few studies have been included, which may lead to bias in the results.

## Discussion

This meta-analysis included seven studies that evaluate the effect of TCS exposure during pregnancy on neonatal birth weight and three studies that evaluate the effect of TCS exposure during childhood on children's BMI. The results showed that there was no significant association between exposure to TCS during pregnancy and neonatal birth weight, as well as exposure to TCS during childhood on children's BMI. Sensitivity analysis showed that after one-by-one elimination, the results were consistent.

Differences in the characteristics of participants, sampling times, and testing methods might lead to different results in the included studies. In the first part of our meta-analysis, Geer et al. (15) and Messerlian et al. (7) found that TCS exposure would decrease neonatal birth weight, while Ouyang et al. (14) found that there was a positive association between maternal TCS and birth weight in female infants, and a non-significant inverse association was found in male infants. These results were different from the conclusions of several other studies (17–20). Moreover, the participants in these two studies of Messerlian et al. (7) and Philippat et al. (18) were predominantly white people from the USA who were older, earned more, and most of them had a higher college education. Calafat et al. (32) found that participants who were white, older, more educated, married, and had a higher household income had higher urinary TCS concentrations, and their infants may have been more affected compared to other women. In Ouyang et al. (14), the BMI of 14.6% of participants was 23–24.9 kg/m^2^, 10.4% of the participants were overweight with a BMI ≥ 25 kg/m^2^, and 12.7% of the participants were diagnosed with gestational diabetes mellitus; we considered that this may lead to the inauthenticity of their results. Furthermore, different studies were adjusted for different potential confounders, and this may also contribute to the inconsistent results. For example, the adjustment variables in Geer et al. (15) were only maternal age group, nativity, and neonate gender, while the adjustment variables of Messerlian et al. (7) were maternal BMI, maternal education, and season (ordinal). On the other hand, the adjustment variables of Ouyang et al. (14) were urinary creatinine, passive smoking, parity, and prepregnancy BMI categories. This may also be the reason why the conclusions of the studies by Geer et al. (15), Messerlian et al. (7), and Ouyang et al. (14) were different from others.

In our second meta-analysis, Li et al. (30) found that TCS exposure was inversely associated with BMI. Buser et al. (29) and Deierlein et al. (31) showed no significant association between TCS and children's BMI. Furthermore, Li et al. (30) detected one spot-urine sample, while other studies detected urine samples multiple times at different gestational weeks, which may have caused some bias because TCS is usually rapidly metabolized and excreted (33). The participants in the studies by Deierlein et al. (31) were girls who came from the USA and were predominantly exposed to TCS during their childhood, while both boys and girls during childhood and adolescence were surveyed in other studies. The difference in age and gender may play key roles in the results. Due to few studies have examined the relationship between children exposure to TCS and BMI by different genders, we could not determine the effect of TCS on BMI in children of different genders.

TCS is an environmental endocrine disruptor with antibacterial activity. Endocrine-disrupting chemicals may alter metabolism *via* estrogenic, antiestrogenic, or antiandrogen action by interfering with other hormone functions (34). However, studies suggest that levels of these chemicals are reasonably stable over time for the purpose of ranking and have acceptable intraindividual variability over more than a year (33). This may explain how exposure to TCS during childhood has no relationship on the children's BMI. Also, exposure to TCS during pregnancy did not effect neonatal birth weight; it was probably due to the placental protection mechanism (35, 36).

Our meta-analysis found that there was no significant association between maternal urinary TCS level during pregnancy and neonatal birth weight, which was consistent with the latest published meta-analysis (37). However, our study had stricter literature inclusion criteria and exclusion criteria. We conducted unified classification and screening according to the types of research data and adjustment methods. To explore the reasons for the existence of heterogeneity, we performed subgroup analysis and sensitivity analysis for the results, while Zhong et al. (37) did not. However, the result of our study differs from the article published in June 2020 (38), which showed that exposure to TCS during pregnancy will increase the birth weight of newborns. However, the CI for the results of the study on birth weight crossed zero in the forest plot, indicating that their results were not statistically significant.

The publication bias of a study by Deierlein et al. (31) was significantly greater than the others. We compared the differences between a study by Deierlein et al. (31), and the other two articles, and found that TCS were adjusted for relatively simple covariates including race, age, educational level, socioeconomic status, and baseline BMI, which may explain the asymmetry of the funnel graph.

The strengths of our study were as follows: First, this meta-analysis analyzed the effects of TCS exposure during pregnancy on children and the effects of TCS exposure in children, respectively. Second, subgroup analyses were performed to explore the relationship between TCS exposure and neonatal birth weight by different adjusted methods. Third, the most relevant studies were high-quality prospective cohort studies, which can enhance the credibility of our study. Fourth, in the first meta-analysis to evaluate the relationship between maternal urinary TCS level and neonatal birthweight, we classified the included studies according to the TCS adjusted methods and performed a meta-analysism. In the other studies, urine TCS concentrations were all adjusted for creatinine, and all of these improved the reliability of the results. Finally, we used birth weight as an indicator and referred to a unified calibration method and unit.

Our study also had limitations. First, birth weight and children's BMI may be influenced by other confounding factors, such as other environmental chemicals, mother or child nutrition, mother or child age, and so on. Inadequate adjustment of confounders might cause overestimation or underestimation of the actual effect of exposure to TCS on outcomes. Second, the studies included were generally from the United States, China, and Europe. There was a lack of relevant research in other regions, which may cause regional biases in this study. Third, the two studies of this meta-analysis contained few records, which may lead to unstable results. What's more, all studies included were log or in the transformation of TCS (considerate the abnormal distribution) except for one study that TCS was not converted.

## Conclusion

In summary, in our meta-analysis, there was no significant association between exposure to TCS during pregnancy and neonatal birth weight, as well as TCS exposure during childhood on children's BMI. We recommend a broader, larger prospective cohort study to assess the relationship between prenatal TCS exposure and neonatal birth outcomes. In addition, we should pay more attention to TCS exposure during childhood and reduce children's exposure to TCS.

## Data Availability Statement

The original contributions presented in the study are included in the article/supplementary material, further inquiries can be directed to the corresponding author/s.

## Author Contributions

JL carefully read and screened the literature related to the research direction and collected the data. DC made figures and tables and they were all the main authors of the manuscript. YH polished the language and optimized the format of the first draft. In the process of reviewing the manuscript, the language was checked and polished by FB. TC reviewed and revised the final manuscript and gave advice during the submission process. XW was the final reviewer. All authors contributed to the article and approved the submitted version.

## Conflict of Interest

The authors declare that the research was conducted in the absence of any commercial or financial relationships that could be construed as a potential conflict of interest.
